# Visual Voices: Hispanic and African American Youth Perspectives on Neighborhood and School Health

**DOI:** 10.3390/children12091165

**Published:** 2025-09-01

**Authors:** Norma Olvera, Rhonda Scherer, Tamal J. Roy, Nelson W. Chavez Cubas, Molly R. Matthews-Ewald, Consuelo Arbona

**Affiliations:** 1 Department of Psychological, Health, & Learning Sciences, University of Houston, 3657 Cullen Boulevard Room 491, Houston, TX 77204-5029, USA; rlschere@central.uh.edu (R.S.); tjroy@cougarnet.uh.edu (T.J.R.); nwchavez@cougarnet.uh.edu (N.W.C.C.); consueloa@central.uh.edu (C.A.); 2 WhitworthKee Consulting, LLC, Washington, DC 20003, USA; mmathewsewald@whitworthkee.com

**Keywords:** health perceptions, school, neighborhood, children, adolescents

## Abstract

**Background/Objectives**: Youth perceptions of their environments provide critical insight into the social and physical determinants of health. This study investigated how Hispanic and African American children conceptualize health within their neighborhood and school settings. **Methods**: A total of 44 participants (29 Hispanic, 15 African American; *Mean age* = 9.27 years, *SD* = 1.28 years) participated in a photovoice project, capturing photographs and quotes in response to two guiding prompts: (1) “*What does your dream of a healthy community look like?*” and (2) “*What should we do to make your community healthier?*” **Results**: Thematic analysis of participants’ quotes and photographs yielded four overarching themes across both neighborhood and school environments: (1) access to nutritious food options, (2) opportunities for physical activity and recreation, (3) clean and safe spaces, and (4) access to extracurricular and community-based programs. A fifth theme, emergency preparedness and response, emerged uniquely within school context. Participants also proposed context-specific solutions to address identified health concerns. **Conclusions**: These findings emphasize the importance of environmental context in shaping child and preadolescent health perceptions and priorities. The results support the development of children and adolescent-informed, community-level interventions aimed at improving environmental health conditions, particularly in underserved communities disproportionately affected by poverty and structural inequities.

## 1. Introduction

Children of families residing in socioeconomically disadvantaged communities are disproportionately exposed to environmental health risks, including lead-based paint, secondhand tobacco smoke, traffic-related air pollution, and a lack of access to health-promoting resources such as green spaces and nutritious foods [1,2]. These exposures increase vulnerability to a range of cognitive, psychological, and physical health comorbidities across the lifespan [3]. According to the US census [4], approximately 11% of the U.S. population, or about 36.8 million individuals, lived below the poverty line in 2023. In addition, race and ethnicity disparities in poverty are stark with 17.8% of Black and 16.6% of Hispanic families living in poverty, compared to 7.7% of White families.

Understanding how children and preadolescents perceive the features of healthy communities is a critical first step in addressing environmental health inequities and informing community-based interventions that promote well-being. A growing body of research highlights the importance of children and adolescents’ perceptions of their neighborhoods and schools in shaping health-related behaviors and outcomes. Regarding eating behavior, Thompson et al. [5] utilized photovoice and conducted interviews with 18 parent–child dyads (56% Black, 44% Hispanic; children aged 8–13 years) to investigate determinants of food choices. Participants identified a range of personal, familial, and environmental influences on their food choices, with children uniquely highlighting the role of school cafeteria offerings and peer behavior. Nabors et al. [6] found that children who photographed family meals increased their awareness of what they ate. In turn, children often expressed a desire for healthier diets at both individual and family levels. According to Hu et al. [7], children and adolescents perceive environmental features such as neighborhood safety and the availability of recreational spaces as relevant factors influencing physical activity levels.

Mental health perceptions are likewise influenced by environmental conditions. For instance, Sprague et al. [8] found that adolescents who were exposed to community violence like hearing gunshots associated these experiences with threats to their safety and psychological well-being. Conversely, having access to green spaces has been shown to be associated with enhanced cognitive functioning and emotional regulation [9], whereas the lack of safe walking routes and green spaces negatively impacted the academic outcomes and general well-being [10].

Although the role of school environments in shaping mental health and academic outcomes has been increasingly examined, the broader concept of health as perceived by children and adolescents within the school context remains underexplored. For example, Aldridge and McChesney [11] conducted a systematic mixed-methods review of 48 studies and identified several school climate factors such as positive peer and teacher relationships, safety, connectedness, and academic support associated with better mental health outcomes. Similarly, Ihlebæk et al. [12] found that students who viewed their schools as inclusive, cohesive, and safe reported improved perceptions of their health. To support this, a quantitative study by Foster et al. [13] discovered that a stronger sense of school belonging was associated with reduced depressive symptoms, lower social anxiety, and higher self-esteem among a racially diverse sample of 224 adolescents (aged 12–15 years; 66.5% female; 52% African American, 29% Caucasian, 16% Multiracial). Hernández et al. [14] also reported that a sense of belonging within the school was positively linked to academic aspirations and performance in a sample of Mexican children and adolescents (*Mean age* = 10.86 years).

Despite these insights, there remains a significant gap in research investigating how children and adolescents of color, particularly Hispanic and African American youth, perceive the health-related aspects of their neighborhood and school environments. Among the few studies focused on these specific demographics, Sprague et al. [8] noted how the photovoice method was a viable approach for predominantly Black preadolescents aged 10 to 12 years to articulate the detrimental effects of trash and pollution, as well as the psychological benefits of clean, green spaces. In this study, participants also proposed solutions at multiple levels, including individual actions such as picking up trash, community efforts like increasing green spaces, and global initiatives such as promoting electric vehicles. Similarly, Kovacic et al. [15] engaged 10 African American children aged 8 to 13 years in documenting health concerns through photographs over a 14-week period, revealing prominent themes such as food insecurity and safety issues. Johnson et al. [16] reported that African American preadolescents aged 10 to 13 years, became increasingly aware of how access to nutritious foods shaped their dietary behaviors and were subsequently motivated to advocate for healthier options after photographing their local food environments. Taken together, these studies demonstrate that photovoice is an appropriate method for Hispanic and African American youth to articulate and document their surroundings.

Despite the efforts to understand children and preadolescents’ perceptions, Hispanic and African American children remain underrepresented in participatory health research that examines children’s understanding and experience of health within their neighborhood and school environments. To address this gap, the present study investigates how Hispanic and African American children and preadolescents perceive the health of their neighborhood and school environments using a photovoice methodology. The following research questions guide this study: (1) How do Hispanic and African American children and preadolescents perceive the health of their neighborhood and school environments? and (2) What strategies do Hispanic and African children and preadolescents suggest for creating healthier communities?

This study is theoretically grounded in the Critical Youth Empowerment (CYE) framework which emphasizes the active involvement of youth in processes of social change, community development, and civic engagement [17]. Jennings et al.’s [17] CYE framework integrates principles from adolescent development and critical pedagogy, highlighting the importance of agency, leadership opportunities, and sociopolitical awareness in fostering youth empowerment. The six key dimensions of CYE include: (1) a welcoming and safe environment, (2) meaningful participation and engagement, (3) equitable power-sharing between youth and adults, (4) engagement in critical reflection on interpersonal and sociopolitical processes, (5) participation in sociopolitical processes to effect change, and (6) integrated individual and community-level empowerment.

Guided by the CYE framework, the present study employed photovoice to explore the health-related perceptions of neighborhood and school environments among Hispanic and African American preadolescents. Photovoice is a participatory qualitative research methodology that engages participants in documenting and critically reflecting on their lived experiences through photography and guiding questions [18]. Central to this method is its ability to facilitate critical dialog, promote collective knowledge production, and serve as a tool for advocacy and social change. Originally developed within the field of public health, photovoice has since been applied across diverse disciplines including education, environmental justice, and youth development [8,19]. Photovoice has demonstrated utility in research involving children and preadolescents, offering a creative and accessible medium through which they can articulate their health perspectives regarding social, environmental, and institutional contexts. This method not only legitimizes youth lived experiences but also positions their voices as essential to informing community transformation and policy development [19,20,21].

## 2. Materials and Methods

### 2.1. Participants

The sample consisted of 44 children and preadolescents (25 girls, 19 boys; 29 Hispanic, 15 African American), with ages ranging from 8 to 12 years (*Mean age* = 9.27 years, *SD* = 1.28 years).

### 2.2. Procedures

This study used archival, deidentified data from children participating in one of two programs. All data (i.e., photovoice and descriptive information) used in this study were deidentified and before data were analyzed, the analysis procedure was reviewed by the referent institution’s Institutional Review Board (IRB) and deemed “exempt” due to the secondary and deidentified nature of the data. Specifically, data were collected from children and preadolescents who participated in one of two separate health programs: (1) a summer wellness program, and (2) an afterschool nutrition/STEM program.

For both programs, the agencies (school and residential community centers) collected parental consent for the children to participate in the afterschool nutrition/STEM programs and summer wellness programs, respectively, which included participation in evaluation activities (e.g., photovoice data). More specifically, program directors recruited participants and obtained parental consent for children and early adolescents to participate in the summer wellness program at a residential community center located within an apartment complex, where residents were predominantly low-income Hispanic (64%) or African American (36%) families.

Participants in the after-school nutrition/STEM program were recruited by a science teacher who obtained parental consent for children and early adolescents to participate in this program. This selected school is from a Title 1 public school serving grades PK-8, with a total enrollment of 1302 students. The ethnicity of the student body enrolled at this school was 90.5% Hispanic, 6.8% African American, and 1.6% White students. Most students (97.8%) were classified as economically disadvantaged. The school was situated in a predominantly Hispanic neighborhood with limited resources; 92% of students were considered at risk of dropping out and 97.8% were considered economically disadvantaged [22].

For both health programs, former research assistants were responsible for designing, implementing, and evaluating a wellness program and a nutrition/STEM afterschool program in 2023. We specifically designed, among other activities, the photovoice activity as a group project for children to express their health perceptions of their neighborhood or school environments. Participants engaged in the photovoice project to express their health perceptions during their participation in a 4-week, 5-day-per-week summer wellness program (summer 2023) or 4-week, twice-weekly after-school nutrition and STEM program (spring and fall 2023). Participants were asked to take photographs of either their neighborhood (summer program) or school (afterschool nutrition/STEM program) in response to two guiding questions about their community health: *(1) “What does your dream of a healthy community look like?”* and *(2) “What should we do to make your community healthier?”* In preparation for the photovoice project, participants for both health interventions attended a 30 min instructional session. During this instructional session, participants received information about the photovoice methodology, were provided sample photographs, and were offered guidance on purposeful photo-taking and explanations for selecting such photos. In addition, participants reviewed examples of photovoice projects to better understand how photography can be used to analyze and communicate community health issues. The following week, research assistants lent each participant a mini-iPad and provided instructions on how to operate it. Notably, approximately 90% of the participants had never used a mini-iPad, iPad, or tablet before. Next, participants were divided into small groups of three to four students, each led by a designated peer leader and supported by a research assistant who facilitated and monitored the group’s photovoice activities.

Following the next session, each group of participants was allotted one hour to take photographs in and around their neighborhood (summer wellness participants) or school (afterschool nutrition/STEM participants). After completing the photo-taking activity, participants reconvened with their group members to share and discuss the images they had taken. During this initial analysis session, participants discussed the meanings of their photographs in relation to the guiding research questions. In the following session, each group selected the images they felt best addressed to these questions. These selected images were shared with the research assistants, who printed copies of the photographs and returned them to each group. Each group then created a visual display by mounting printed photographs on a poster board along with their corresponding quotes for each respective photograph that was selected and offered solutions to address the participants’ health concerns. The poster boards served as the centerpiece for a final group presentation, and participants shared their insights regarding solutions to address their health concerns with their peers. Given the relevant qualitative and visual data generated through the photovoice approach, we included preliminary analyses of the photovoice data in the required reports submitted to the science schoolteacher and principal, or residential community center directors for whom the program evaluations were conducted. For the present study, the current research team conducted a more systematic and in-depth analysis of the original qualitative and visual photovoice data.

#### Statistical Plan

An inductive thematic analysis was conducted to examine participants’ verbatim narratives and photographs, enabling themes to emerge directly from the data in accordance with established qualitative research practices [23]. This method is particularly well-suited for exploring lived experiences and social phenomena, where it allows for a nuanced understanding of themes that are grounded in participants’ perspectives. The trustworthiness of the findings was established through multiple strategies: independent coding, iterative peer debriefing, and triangulation of narrative and visual data. More specifically, the four-person coding team independently reviewed the final selections of photographs and quotes from the groups, noting initial impressions and developing preliminary thematic categories. The photographs taken by participants served as elicitation tools to validate participants’ quotes. Then, the coders reviewed photographs along with the quotes to independently generate the initial themes. Coding was recorded digitally using Excel spreadsheets, allowing for real-time updates and efficient comparison across coders. Initial codes were compared collaboratively among the team and lead investigators to refine themes and ensure analytic consistency. Weekly team meetings were held to reconcile discrepancies, discuss interpretations, and ensure coherence across coding decisions. The iterative, consensus-driven process enhanced the reliability and validity of the thematic framework, aligning with best practices in qualitative analysis [23]. This process ensured that the final themes accurately reflected children and adolescents’ perceptions of neighborhood and school health, with themes being refined through continuous comparison and validation.

## 3. Results

### 3.1. Sample Descriptive Characteristics

The original number of participants consisted of 60 children and preadolescents. However, for this study, only participants who attended at least 50% of the programmatic sessions for either the summer or after-school programs were included in the qualitative analysis to ensure that participants had sufficient engagement with the program and meaningful involvement in the group-based photovoice project. Thus, the final sample for this study was 44 participants. Of the 44 total participants, 9 participants (1 Hispanic girl, 7 African American girls, and 1 African American boy) completed the photovoice project during the summer program. The remaining 35 participants (15 Hispanic girls, 13 Hispanic boys, 2 African American girls, and 5 African American boys) participated in the photovoice project as part of the afterschool nutrition/STEM program.

### 3.2. Description of Themes

Following thematic analysis of the participants’ quotes and photographs, four overarching themes emerged regarding health in neighborhood and school environments. (See Table 1 for summary of the themes and representative photographs, and suggested solutions. These themes included having access to (1) nutritious food options, (2) physical activity and recreation, (3) clean and safe spaces, and (4) extracurricular and community-based programs. An additional theme, emergency preparedness and response, arose regarding the school’s health environment.

#### 3.2.1. Theme 1: Access to Nutritious Food Options

Participants’ quotes and photographs revealed a desire for improved access to nutritious food options in their neighborhood and school contexts. The participants in the summer wellness program emphasized the need for larger and more comprehensive community gardens (see the photograph of a community garden in Table 1 under the neighborhood category), as well as access to fresh produce at the local grocery stores. As a participant group shared, “*Health is having a store with healthy food*,” underscoring the importance of having access to healthy food in the neighborhood. At the school level, participants who attended the afterschool nutrition/STEM program focused on the importance of having access to nutritious food (e.g., access to school vegetable gardens, larger cafeterias, and buffet-style service with healthy food choices) and clean water at the school. For example, filtered water stations were frequently photographed throughout the school (Table 1 has a photograph of water fountains), with participants noting “*It makes the water better*,” and emphasized the value of “*free water to kids*.” Children linked access to clean water not only to drinking water but also to the ability to grow nutritious foods in the garden. Overall, participants connected health with access to clean water, green spaces, and nutritious food, advocating for improvements in both environments.

#### 3.2.2. Theme 2: Physical Activity and Recreation

Participants from both programs consistently highlighted the importance of physical activity to promote health through images of parks, open fields, and playgrounds. In neighborhoods, participants in the summer program expressed a need for better access to recreational spaces such as basketball courts, football fields, playgrounds, jungle gyms, and community pools to be active. Open space was valued for its role in promoting physical activity and energy release. Within the school environment, participants advocated for larger gyms, more sports fields, increased physical activity during recess, and shaded outdoor playgrounds. Table 1 features outdoor recreation spaces such as a jungle gym at an apartment complex in the neighborhood and a playground that was photographed in the school setting. Participants from the afterschool nutrition/STEM program remarked, “*We need more covered space to play outdoors*,” indicating a desire for weather protected outdoor activity areas. Table 1 also includes solutions that underscore participants’ concerns related to fixing uneven parking lot pavements, broken benches, and broken garden doors at the neighborhood level. Overall, participants from both programs emphasized that access to safe, stimulating, and shaded areas for physical activity was central to their concept of a healthy community, both in the neighborhood and school environments.

#### 3.2.3. Theme 3: Access to Clean and Safe Neighborhoods and Schools

Participants in the summer wellness program took photographs of their neighborhoods that frequently depicted trash and litter in shared spaces like sidewalks, parking lots, and green areas. Sometimes this litter included hazardous items like drug paraphernalia and harmful substances. They also voiced concerns about broader safety issues such as the presence of weapons, crime, unsafe transportation and infrastructure concerns regarding reliable internet connection and a functioning electrical gate in their apartment complex. Students from the summer wellness program reported being exposed to “*Cloudy water that smells unsanitary and close to electricity grid*”. In school settings, cleanliness was likewise also a key concern. Students noted that excessive trash in classrooms, cafeterias, and playgrounds undermined the image of a healthy school environment. Participants in the school settings commented, “*We can be cleaner at school*,” emphasizing the collective responsibility for maintaining a healthy environment. There are photographs of littering within neighborhood and school settings in Table 1 as well as proposed solutions to address litter by, for example, placing more trash cans in school hallways and enforcing no-littering policies at the neighborhood level. Overall, participants expressed a strong desire for both clean and safe spaces where they could learn and play without fear, highlighting their awareness of how environmental conditions affect health and well-being.

#### 3.2.4. Theme 4: Extracurricular and Community-Based Programs

Participants in both programs expressed appreciation for natural environments and extracurricular programs that support mental and emotional health. In neighborhood settings, the students in the summer wellness program valued green spaces, shaded areas, and gardens for relaxation and play. Participants stated, “*This is a good representation of a healthy community. I see lots of trees and grass*.” Participants within the afterschool nutrition/STEM program photographed and discussed arts and music programs. Participants took pride in resources like pianos and guitars. Some expressed that “*Our school has pianos and other schools don’t.*” Flyers and posters advertising extracurricular activities were also photographed, showing that participants valued access to enrichment opportunities. Table 1 showcases the images of open playground spaces that participants for their neighborhood and the photographs of pianos which the participants in the after-school program frequently admired. These activities were associated with joy, self-expression, and emotional well-being, which are important elements of a healthy school experience.

#### 3.2.5. Theme 5: Emergency Preparedness and Response (School Only)

Participants in the afterschool nutrition/STEM program also specifically highlighted the importance of having emergency safety measures in place at school. A photograph of the Automated External Defibrillators (AEDs) is included in Table 1. Participants explained, “*We chose this photo because it keeps people safe*,” referencing a photograph of an AED. Others in the afterschool program emphasized the need for clear emergency plans: “*It is important to know what to do in case of an emergency like fire*.” These responses show that students in the afterschool setting not only recognized the value of life-saving resources but also appreciated the importance of being prepared for emergencies. Table 1 includes participants’ proposed solutions to have more emergency drills and resources for potentially dealing with other health emergency situations. Their emphasis on safety and readiness reflects a nuanced understanding of what contributes to a healthy school environment.

## 4. Discussion

When young people are included in decision-making about health, they feel empowered and responsible, ultimately becoming active contributors to their community’s well-being. This study offers valuable insight into the health perceptions of Hispanic and African American children and preadolescents living in underserved neighborhoods who attend under-resourced schools. This research amplifies the voices of marginalized youth by shedding light on their lived experiences, health concerns, and recommendations for changes in their communities. Previous research supports the idea that children and preadolescents’ involvement in community health initiatives leads to sustainable solutions and strengthens community ties. For example, Isma et al. [24] and Kontak et al. [25] emphasize that meaningful and intentional children and adolescent engagement in school health promotion increases the youth’s sense of well-being. In addition, our findings regarding the relevance of having access to nutritious food and safe spaces for engaging in physical activity as essential components of youth health are consistent with previous studies [1,26]. However, disparities in the availability of these resources in low-income and underserved communities exacerbate existing health inequalities and contribute to poor dietary habits and sedentary behaviors among children and adolescents [27]. Thus, implementing neighborhood and school-based programs that promote physical activity, healthy eating, and mental well-being may be instrumental in reducing health disparities among children and adolescents in underserved communities [28].

A central theme identified in this study was the need for access to nutritious food in both school and neighborhood settings. Consistent with Thompson and colleagues’ study [5], findings from the current study indicate that participants recognized the school contexts as an influential factor affecting eating behaviors positively (e.g., providing access to healthy foods). The present study goes further in suggesting specific strategies that schools can implement to promote healthy eating such as having a school vegetable garden and larger cafeterias. Furthermore, findings from the current study expand on our previous work by examining neighborhood influences on eating behavior. Unlike the Thompson and colleagues’ study [5] which focused on the home (e.g., having healthy foods in the home) as an environmental factor influencing their eating behaviors, the present study highlights the broader neighborhood factors such as having access to fresh food at grocery stores and access to bigger community gardens to foster healthy eating. Further, Thompson et al. [5] primarily focused on the nutritional aspects of health, whereas the present study highlights the importance of physical activity, clean and safe environments, access to extracurricular activities, and preparedness response to promote healthy communities. Findings from this study are also consistent with other research [16] that found that urban African American children participating in a photovoice project report limited access to healthy food in their communities as barrier to healthy eating. Furthermore, our findings bolster Hanemaayer et al. [29]’s results regarding structural barriers limiting food access for Indigenous youth in urban settings and reinforcing the importance of community-driven solutions.

Participants in the current study emphasized the importance of opportunities for physical activity in both the neighborhood and school settings. More specifically, they advocated for improved gym facilities, more sports fields, and structured extracurricular programming, in both their immediate neighborhoods and schools. This mirrors findings in earlier research that highlighted the benefits of physical activity on children’s physical health, mental well-being, and academic performance [30], and emphasizes the critical role of urban planning in promoting active living spaces, especially in underserved areas [31]. Creating safe, accessible parks and pedestrian-friendly infrastructure is imperative for encouraging physical activity among children and adolescents in these communities.

The theme of a disproportionate lack of safe, accessible recreational spaces in low-income neighborhoods contributes to sedentary behavior and elevated rates of childhood obesity [3,32]. This theme also aligns with previous research indicating that children are highly sensitive to the conditions of their physical surroundings, which can also impact both their mental and physical health. Importantly, Ihlebæk et al. [12] found that children’s perceptions of school safety and inclusion are strongly associated with self-reported health. Addas [33] similarly identified the accessibility of recreational facilities and neighborhood safety as crucial factors in promoting physical activity among children and adolescents.

Participants across both the school and neighborhood environments consistently identified environmental cleanliness and safety as vital components of health. More specifically, they highlighted specific areas such as streets, playgrounds, classrooms, and cafeterias as needing improvement. Participants described trash-filled public areas and deteriorating infrastructure as major concerns in both neighborhood and school settings. They also expressed concerns about the presence of weapons and unsafe water. These findings align with Xiao et al. [34] and Trask et al. [35], who highlight the negative health impacts of poor environmental conditions on children in disadvantaged communities.

Children and preadolescents in the current study also advocated for cleaner school spaces and emphasized a shared responsibility for maintaining a healthy learning environment. Finally, participants recognized the importance of natural environments for supporting mental and emotional well-being. They advocated for more green spaces, shaded areas, and gardens for play and relaxation. Taken together, these findings support previous research that documented the mental health benefits of exposure to urban green spaces among children and adolescents including enhancing happiness, physical health, social cohesion, and overall well-being [2,31,36].

A unique theme that emerged from the school setting was the value placed on emergency preparedness. Participants photographed Automated External Defibrillators (AEDs) and emergency procedures, emphasizing the importance of safety in the school environment. This reflects a deeper need for environments where children and adolescents feel physically and emotionally safe, which is fundamental for their overall health and development [12].

The inequities highlighted in this study do not occur in a vacuum and are instead deeply rooted in historical and ongoing forms of structural racism, including redlining, disinvestment in communities of color, and discriminatory zoning policies. For example, the participants’ concerns about access to fresh produce items, safety hazards, and deteriorating infrastructure reflect the cumulative effects of systemic exclusion from health-promoting environments. Singleton et al. [37] found that all 21 studies in their scoping review reported statistically significant associations between reduced access to food retailers and redlining, segregation, and racialized disinvestment. Similarly, Li and Yuan [38] reported that historically redlined neighborhoods continue to experience limited access to greenspace and supermarkets across U.S. cities. These findings reflect broader structural patterns that restrict health-promoting resources in communities of color. The Woods-Jaeger et al. [39] study highlights how centering youth of color in health equity efforts advances structural change, which aligns with the CYE framework. Naming these forces explicitly positions youth not just as observers of inequity, but as potential agents of structural transformation. In this study, participants’ recommended solutions were included in a final report provided to stakeholders (e.g., school science teacher and school principal or residential community center directors).

While this study provides valuable insights into the health perceptions of neighborhood and school environments among Hispanic and African American children and preadolescents, several limitations should be acknowledged. First, there may have been a selection bias, given that the sample was limited to Hispanic and African American children and preadolescents participating in healthy intervention programs in low-income communities. In addition, only children who attended at least 50% of the programmatic sessions were included in the current study. Furthermore, we acknowledge the differences in sample sizes of participants whose data were collected during the summer wellness program (9 participants) and nutrition/STEM program (35 participants). These factors may have resulted in the final sample not representing the broader experiences of Hispanic and African American children and preadolescents from other socio-economic or cultural backgrounds. Future research could include a more diverse population of youth of color to examine whether similar themes emerge across different contexts. For example, studies could investigate how cultural values, community resources, and local policy interventions affect the health behaviors and perceptions of children from diverse ethnic backgrounds. Additionally, future studies should examine the long-term impact of environmental changes in neighborhoods and schools on the health perceptions and outcomes of children, especially in underserved communities.

We would also like to recognize the constraints of the data collection procedure itself such as allotting participants only one hour to take photographs in their neighborhood or school. This limited timeframe inherently curtails the geographic scope of what could be documented and may have prevented the capture of environmental aspects that may only be apparent at different times of day (e.g., evening safety concerns or after-hours use of public spaces). Another limitation of this study was our inability to assess how gender may have shaped youth health perceptions of their neighborhood and school environment because we collected data at an aggregated level. To address these limitations, future studies should allow more time for youth of color to take pictures as well as assessing gender differences in their health perceptions of their neighborhood and school environments.

## 5. Conclusions

Despite the aforementioned limitations, this study contributes to the growing, yet relatively sparse, body of research on children and preadolescent health perceptions in underserved environments, with a specific focus on Hispanic and African American children and preadolescents. By using photovoice methodology, the current study not only captured the perspectives of marginalized children and preadolescents but also empowered them to actively engage in the process of advocating for improvements in their local communities. The findings emphasize the critical role of clean environments, access to physical activity, and nutritious food in shaping children’s health perceptions. Ultimately, this research highlights the need for targeted interventions that address the environmental factors influencing health outcomes among low-income children of color, with the goal of improving the overall well-being of future generations. One mean by which to identify ways to address environmental impacts is through engaging the youth in solutioning. In support of this, Malorni et al. [40] stresses the value of youth participatory action research (YPAR) in promoting relational practices that empower children and adolescents to contribute to community health efforts. Ensuring that youth can engage in solutioning not only address immediate environmental concerns but also cultivates long-term community engagement and youth agencies.

## Figures and Tables

**Table 1:** Category, Photos, Health Concerns, and Themes.

Category	Photo	Specific Health Concern	Theme
**Neighborhood**	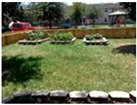	▪ Access to fresh food at grocery stores ▪ Access to bigger community gardens	Access to Nutritious Food Options
	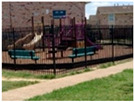	▪ Basketball courts ▪ Football fields ▪ Community pools ▪ Playgrounds ▪ Clubhouse ▪ Exercise/Activity in nature ▪ Access to clean water while exercising ▪ Gardens	2. Opportunities for Physical Activity and Recreation
	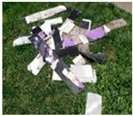	▪ Clean complex apartments and parking ▪ Clean streets ▪ Better weapon control ▪ Better law enforcement ▪ Safe transportation	3. Clean and Safe Spaces
	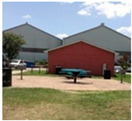	▪ Neighborhood ▪ Service Programs ▪ Youth Programs ▪ Access to free internet	4. Access to Extracurricular and Community-Based Programs
**Proposed Solutions**: Neighborhood level: Participants proposed solutions for reducing litter and improving green areas and infrastructure in their apartment complex. **Specific solutions included**: **Littering Reduction** Use fewer plastic water bottles. Have residents pick up trash and have better services programs to keep a “clean environment.” Speak to the apartment management about enforcing no littering rules, establishing a volunteer day for residents to pick up trash, and making more trash cans available. **Green Areas Improvements** Mow the grass more often, add more grass areas to play with, water grass more often, and add more flowers. **Infrastructure Improvements** Repair uneven parking lot pavement, broken garden doors, broken benches, and broken electrical gate immediately to operate correctly. Lastly, participants suggested fundraising such as seeking individual donations and having raffles, car washes, garage sales, bake and lemonade sales, and toy sales.			
**School**	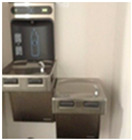	▪ Access to clean water ▪ Access to a vegetable garden ▪ Access to a bigger cafeteria ▪ Access to a buffet healthy food options	Access to Nutritious Food Options
	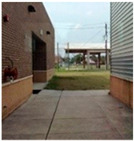	▪ Access to facilities to practice sports ▪ Access to basketball courts to play ▪ A bigger gym ▪ Fields for basketball and soccer ▪ Field trips to a soccer stadium ▪ Access to outdoor shaded areas ▪ More outdoor covers to make more space ▪ Access to a game room ▪ A water park ▪ Offer swimming and yoga classes	2. Opportunities for Physical Activity and Recreation
	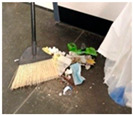	▪ Have clean classrooms ▪ Access to clean cafeteria, not littering ▪ Access to clean outdoor areas to exercise	3. Clean and Safe Spaces
	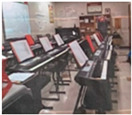	▪ Need for extracurricular programs that increase art skills and dance skills. ▪ Need for advertising extracurricular activities on informational bulletin boards.	4. Access to Extracurricular and Community-Based Programs
	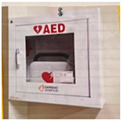	▪ Importance of having emergency devices at school, such as the use of Automated External Defibrillators ▪ Need to have emergency trials	5. Emergency Preparedness and Response
**Proposed Solutions:** School level: Participants proposed solutions for increasing healthy eating, physical activity, clean schools, and school health policies. **Specific solutions included**: **Healthy Eating Promotion** Offer healthier food choices at the cafeteria, have school gardens and add benches to sit down in the garden, and have bigger water fountain filters. **Physical Activity Promotion** Provide more shady areas in the playground. Offer other physical activities such as swimming as part of physical education. **Clean Schools** Place more trash cans in the hallways so participants can “*throw away the trash as we see*”. **School Policy Change Recommendation** Increase the allowed time for students to use the restroom.			

## Data Availability

The data are not publicly available due to concerns regarding privacy.

## References

[1] Manduca R., Sampson R.J. (2019). Punishing and Toxic Neighborhood Environments Independently Predict the Intergenerational Social Mobility of Black and White Children. Proc. Natl. Acad. Sci. USA.

[2] Yoon J., Lee C. (2019). Neighborhood Outdoor Play of White and Non-White Hispanic Children: Cultural Differences and Environmental Disparities. Landsc. Urban Plan..

[3] Kirkbride J.B., Anglin D.M., Colman I., Dykxhoorn J., Jones P.B., Patalay P., Pitman A., Soneson E., Steare T., Wright T. (2024). The Social Determinants of Mental Health and Disorder: Evidence, Prevention and Recommendations. World Psychiatry.

[4] US Census Bureau (2024). Poverty in the United States: 2023.

[5] Thompson D., Callender C., Velazquez D., Adera M., Dave J.M., Olvera N., Chen T.-A., Goldsworthy N. (2021). Perspectives of Black/African American and Hispanic Parents and Children Living in under-Resourced Communities Regarding Factors That Influence Food Choices and Decisions: A Qualitative Investigation. Children.

[6] Nabors L., Murphy M.J., Lusky C., Young C.-J., Sanger K. (2020). Using Photovoice to Improve Healthy Eating for Children Participating in an Obesity Prevention Program. Glob. Pediatr. Health.

[7] Hu D., Zhou S., Crowley-McHattan Z.J., Liu Z. (2021). Factors That Influence Participation in Physical Activity in School-Aged Children and Adolescents: A Systematic Review from the Social Ecological Model Perspective. Int. J. Environ. Res. Public Health.

[8] Sprague N.L., Zonnevylle H.M., Jackson Hall L., Williams R., Dains H., Liang D., Ekenga C.C. (2023). Environmental Health Perceptions of Urban Youth from Low-Income Communities: A Qualitative Photovoice Study and Framework. Health Expect..

[9] Ly V., Vella-Brodrick D.A. (2024). Effects of School-Led Greenspace Interventions on Mental, Physical and Social Wellbeing in Children and Adolescents: A Systematic Review. Educ. Psychol. Rev..

[10] Bates C.R., Bohnert A.M., Gerstein D.E. (2018). Green Schoolyards in Low-Income Urban Neighborhoods: Natural Spaces for Positive Youth Development Outcomes. Front. Psychol..

[11] Aldridge J.M., McChesney K. (2018). The Relationships between School Climate and Adolescent Mental Health and Wellbeing: A Systematic Literature Review. Int. J. Educ. Res..

[12] Ihlebæk C., Castellan C., Flobak J., Ese J. (2021). The School as an Arena for Co-Creating Participation, Equity, and Well-Being-A Photovoice Study from Norway. Int. J. Environ. Res. Public Health.

[13] Foster C.E., Horwitz A., Thomas A., Opperman K., Gipson P., Burnside A., Stone D.M., King C.A. (2017). Connectedness to Family, School, Peers, and Community in Socially Vulnerable Adolescents. Child. Youth Serv. Rev..

[14] Hernández M.M., Robins R.W., Widaman K.F., Conger R.D. (2016). School Belonging, Generational Status, and Socioeconomic Effects on Mexican-Origin Children’s Later Academic Competence and Expectations. J. Res. Adolesc..

[15] Kovacic M.B., Stigler S., Smith A., Kidd A., Vaughn L.M. (2014). Beginning a Partnership with PhotoVoice to Explore Environmental Health and Health Inequities in Minority Communities. Int. J. Environ. Res. Public Health.

[16] Johnson K.A., Steeves E.A., Gewanter Z.R., Gittelsohn J. (2017). Food in My Neighborhood: Exploring the Food Environment through Photovoice with Urban, African American Youth. J. Hunger Environ. Nutr..

[17] Jennings L.B., Parra-Medina D.M., Hilfinger-Messias D.K., McLoughlin K. (2006). Toward a Critical Social Theory of Youth Empowerment. J. Community Pract..

[18] Wang C., Burris M.A. (1997). Photovoice: Concept, Methodology, and Use for Participatory Needs Assessment. Health Educ. Behav..

[19] Liebenberg L. (2022). Photovoice and Being Intentional about Empowerment. Health Promot. Pract..

[20] Leung M.M., Agaronov A., Entwistle T., Harry L., Sharkey-Buckley J., Freudenberg N. (2017). Voices through Cameras: Using Photovoice to Explore Food Justice Issues with Minority Youth in East Harlem, New York. Health Promot. Pract..

[21] Abma T.A., Schrijver J. (2020). ‘Are We Famous or Something?’ Participatory Health Research with Children Using Photovoice. Educ. Action Res..

[22] Murphy R., Daniel A., Cai M., Lau E., Schumacher Y., Astudillo C., Reid R. Pilgrim Academy. Texas Public Schools. https://schools.texastribune.org/districts/houston-isd/pilgrim-academy/.

[23] Braun V., Clarke V. (2006). Using Thematic Analysis in Psychology. Qual. Res. Psychol..

[24] Isma G.E., Rämgård M., Enskär K. (2023). Perceptions of Health among School-Aged Children Living in Socially Vulnerable Areas in Sweden. Front. Public Health.

[25] Kontak J.C., Caldwell H.A.T., Kay-Arora M., Hancock Friesen C.L., Kirk S.F.L. (2022). Peering in: Youth Perspectives on Health Promoting Schools and Youth Engagement in Nova Scotia, Canada. Health Promot. Int..

[26] Zhang Y., Mavoa S., Zhao J., Raphael D., Smith M. (2020). The Association between Green Space and Adolescents’ Mental Well-Being: A Systematic Review. Int. J. Environ. Res. Public Health.

[27] Showell N.N., Cole K.W., Johnson K., DeCamp L.R., Bair-Merritt M., Thornton R.L.J. (2017). Neighborhood and Parental Influences on Diet and Physical Activity Behaviors in Young Low-Income Pediatric Patients. Clin. Pediatr..

[28] Francis L., DePriest K., Wilson M., Gross D. (2018). Child Poverty, Toxic Stress, and Social Determinants of Health: Screening and Care Coordination. Online J. Issues Nurs..

[29] Hanemaayer R., Anderson K., Haines J., Lickers K.R., Lickers Xavier A., Gordon K., Tait Neufeld H. (2020). Exploring the Perceptions of and Experiences with Traditional Foods among First Nations Female Youth: A Participatory Photovoice Study. Int. J. Environ. Res. Public Health.

[30] Jaekel J. (2024). The Role of Physical Activity and Fitness for Children’s Wellbeing and Academic Achievement. Pediatr. Res..

[31] Zhang X., Warner M.E. (2023). Linking Urban Planning, Community Environment, and Physical Activity: A Socio-Ecological Approach. Int. J. Environ. Res. Public Health.

[32] Suglia S.F., Campo R.A., Brown A.G.M., Stoney C., Boyce C.A., Appleton A.A., Bleil M.E., Boynton-Jarrett R., Dube S.R., Dunn E.C. (2020). Social Determinants of Cardiovascular Health: Early Life Adversity as a Contributor to Disparities in Cardiovascular Diseases. J. Pediatr..

[33] Addas A. (2025). Impact of Neighborhood Safety on Adolescent Physical Activity in Saudi Arabia: Gender and Socio-Economic Perspectives. Front. Public Health.

[34] Xiao Y., Mann J.J., Chow J.C.-C., Brown T.T., Snowden L.R., Yip P.S.-F., Tsai A.C., Hou Y., Pathak J., Wang F. (2023). Patterns of Social Determinants of Health and Child Mental Health, Cognition, and Physical Health. JAMA Pediatr..

[35] Trask S., D’Souza E.N., Pi S., Tu’akoi S., Bay J.L. (2024). Promoting School-Based Learning about Nutrition and Physical Activity Using Photovoice: A Systematic Review. Health Educ. J..

[36] Syamili Takala T., Korrensalo A., Tuittila E.-S. (2023). Happiness in Urban Green Spaces: A Systematic Literature Review. Urban For. Urban Green..

[37] Singleton C.R., Wright L.A., McDonald M., Archer I.G., Bell C.N., McLoughlin G.M., Steeves E.A. (2023). Structural Racism and Geographic Access to Food Retailers in the United States: A Scoping Review. Health Place.

[38] Li M., Yuan F. (2022). Historical Redlining and Food Environments: A Study of 102 Urban Areas in the United States. Health Place.

[39] Woods-Jaeger B., Jahangir T., Lucas D., Freeman M., Renfro T.L., Knutzen K.E., Lightfoot A.F. (2024). Youth Empowered Advocating for Health (YEAH): Facilitating Partnerships Between Prevention Scientists and Black Youth to Promote Health Equity. Prev. Sci..

[40] Malorni A., Lea C.H., Richards-Schuster K., Spencer M.S. (2022). Facilitating Youth Participatory Action Research (YPAR): A Scoping Review of Relational Practice in U.S. Youth Development & out-of-School Time Projects. Child. Youth Serv. Rev..

